# Magnetic resonance imaging and computerised tomography findings in an intraspinal extradural hydatid cyst mimicking tuberculous spondylitis: a case report

**DOI:** 10.4076/1757-1626-2-7109

**Published:** 2009-06-25

**Authors:** Long Xin, Zhenbin Wang, Shunwu Fan

**Affiliations:** 1 Orthopaedic laboratory, Clinic Medical Research Institution, Sir Run Run Shaw Hospital, Zhejiang University School of MedicineNo.3 Qingchun Road, Hangzhou, 310016, Zhejiang ProvinceChina; 2 Department of Orthopaedics, The Fourth Affiliated Hospital, Xinjiang Medical UniversityXinjiang, 830002China

## Abstract

Spinal hydatid cyst with thoracic vertebra involvement is rare but serious condition. We present a 63-year old woman with spinal hydatid disease mimicking tuberculous spondylitis. A case study with Computerised Tomography and Magnetic Resonance Imaging diagnostic findings and surgical treatment is reported in this article. Primary spinal hydatid disease should be considered in the differential diagnosis of tuberculous spondylitis in endemic area. Familiarity with typical imaging appearances of spinal hydatid disease may be helpful in making a correct diagnosis and treatment.

## Introduction

Cystic hydatid disease, a worldwide zoonosis, is a serious parasitic infection in endemic areas inhabited by carriers of *Echinococcus granulosus*, that is, dogs and livestock (e.g., cattle, goats, and sheep). Humans contact the disease from direct contact with the infected animal or its feces or via contaminated food [1,2]. Hydatid cysts formed in livers and lungs are common, while hydatid cysts formed in spine account for 1% of all cases of hydatid disease [3,4]. Furthermore bone involvement is seen in only 0.5 2% of cases [5]. Thoracic vertebral involvement is extremely rare.

Tuberculous spondylitis is usually secondary to hematogenous dissemination from a pulmonary source, and typically involves both the vertebral body and the adjacent paravertebral tissue. The disease presents insidiously with back pain, and it also presents with spinal cord dysfunction due to epidural compression. It is most commonly localized in the thoracic portion of the spine. The infection first appears adjacent to a disc space and spreads to the rest of the vertebral body. Destruction of vertebrae resulting in curvature of the spine is quite characteristic.

Clinical and imaging findings of the two diseases are hard to distinguish. We here report the rare case of a patient with intraspinal extradural hydatid cyst in the T11 vertebral body and involvement in paravertebral tissue. The purpose of this article is to provide some findings in spinal hydatid cyst to be helpful in making a correct diagnosis and treatment in endemic area.

## Case presentation

A 63-year-old woman from Xinjiang province of western China, who had not suffered from any other disease till then, presented with back pain, progressive weakness, and numbness in both legs and difficulty in walking. All symptoms appeared 8 months earlier.

Physical examination revealed a spastic paraparesis and hypoesthesia below L1. The abdominal skin and cremasteric reflexes were absent and Babinski's sign was positive bilaterally, with bilateral Achilles tendon clonus.

**Figure 1:**
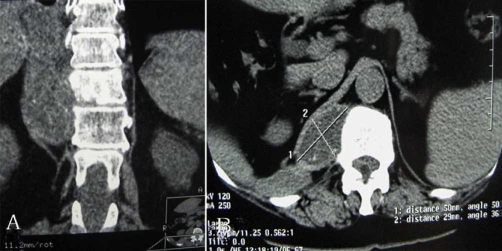
**(A)** Coronal CT scans showed that right lateral of paraspinal cystic lesions were involved in partial vertebra at the level of T9 12. The prominent destruction of T11 vertebra was visible. **(B)** Axial CT scans at the level of T11 showed a mass of 5 × 2.9 cm diameter was localized in the posterior mediastinum. The cyst has water density and at the posterior wall there are some foci of calcification.

**Figure 2:**
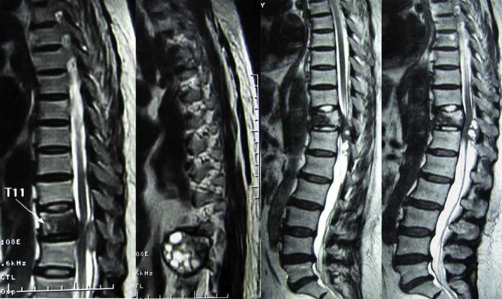
Sagittal T2-weighted MR images showed paraspinal cystic lesion was involved in T11 vertebrae and disc space. Multiple small daughter cysts formed a grape bunch-like image. The thoracic dural sac was narrowed due to the compression of multiple small hydatid cysts like CSF signal intensity.

**Figure 3:**
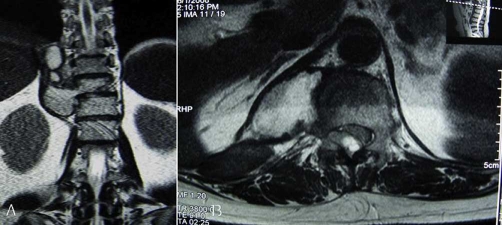
**(A)** Coronal MR images showed paravertebral lesion had expanded to the T11 neural foramina. **(B)** Axial T2-weighted MR images revealed spinal cord was compressed due to the multilocular hydatid cyst extended into the spinal canal. The main part of the lesions was located at the right side of T11.

A coronal thoracic CT scans revealed that the lytic lesion with vertebral destruction had extended to the paravertebral tissue of T9 T12. A cystic mass of 5 × 2.9 cm diameter was located in the posterior mediastinum at the level of T11. The cyst had water density and there were some foci of calcification at the posterior wall (Figure 1a b).MRI of the thoracic region revealed the lesion had extensively involved the T11 vertebrae and disc space. On sagittal T2-weighted images, T11 12 disc space seemed to be decreased and multiloculated scoleces extended into the para-vertebral region. These scoleces, which formed a resemblance to bunch of grapes in patches, also extended toward the spinal canal and affected the extradural space (Figure 2).Coronal MRI image showed the lesion involvement in paravertebral tissue had expanded to the T11 neural foramina. The spinal cord was excessively compressed by several small daughter cysts like cerebrospinal fluid (CSF) signal intensity in the extradural space (Figure 3a b). Serological test was negative.

**Figure 4:**
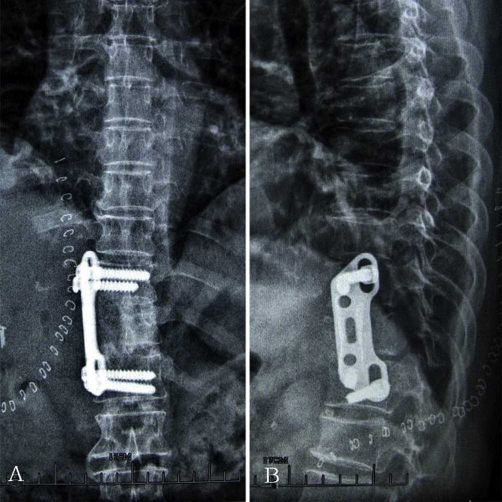
**(A) & (B)** Anteroposterior and lateral radiograph of thoracic vertebra showed anterolateral decompression and fusion was performed on T10 12.

Since the presence of a neurological deficit was clear, the patient was operated on anterior circumferential decompression. All the cysts were removed and then the cavity was irrigated with hypertonic saline. Anterolateral vertebrectomy, fusion and fixation were performed between T10 12 with an iliac autograft (Figure 4a b). Histopathological examination revealed a hydatid cyst.

Albendazole treatment (400 mg/day) was applied in the early postoperative stage. The patient was successfully treated with five cycles of albendazole administered intermittently in 4-week courses followed by 10-day drug-free intervals. During follow up, paraparesis and hypoesthesia was improved almost completely over one year.

## Discussion

Hydatid disease has a worldwide distribution and causes health problems in endemic countries, including china. The disease most often affects liver and lung. Only 0.5-2% is located in the skeletal system,and in approximately 50% of these cases the spinal column is involved [6,7]. Spinal hydatid cysts are usually situated in the dorsal region and generate medullary or radicular symptoms according to their location [3,8,9]. Primary intraspinal extradural hydatid cysts are very rare.

Primary hydatid disease suggests that the parasite's embryo is possibly being carried through the porto-vertebral venous shunts. The growth of hydatid cyst occurs along the intratrabecular spaces with small diverticulated cysts that are formed by exogenous vesiculation [10]. Enlargement and spread of hydatid cyst may result in local erosion of bone. Finally, Pain, deformity, and weakness may result from either collapse of the spine or by extension of hydatid cysts into the spinal canal [6,11]. Neurological deterioration is usually very slow, but will result in paraplegia in 25-50% of cases [12].

Spinal hydatid disease may easily be confused with tuberculous spondylitis in some areas where tuberculosis is endemic. A typical characteristic of tuberculous spondylitis is in paradiscal lesion with disc-space narrowing. Paraspinal extension is very common, with calcification in the mass being pathognomonic for tuberculous infection. Hydatid disease usually involved in the thoracic and lumbar regions where are also typical locations for tuberculous spondylitis. Therefore, the two entities may mimic each other, making differential diagnosis difficult.

MRI may show important differences and aid in early diagnosis and treatment. MRI imaging revealed precise anatomic localization and extension of the spinal hydatid disease. In this case, Cysts had thin walls and CSF-like signal intensity on MR images. On T2-weighted images, cyst scoleces appeared more hyperintense, whereas small vesicles and daughter cysts were visible in a bunch-of-grapes pattern. MRI showed the cysts had a liquid component tendency to invade anatomical cavities through the neural foramen. CT scanning may be more convenient and more advantageous in following the progress of bone lesions associated with this disease. Although plain radiographs can show in the advance stage the bone destruction, the radiological features are not pathognomonic [13]. This case suggested that the differential diagnosis may be preferred on MR images because of the multicystic nature of the disease. CT scanning provided a precise assessment of the osseous part of the lesion and the calcifications of the cyst. And MRI was the superior method in the diagnosis in involvement of neural structures, extension into the soft tissues. Consequently, CT and MRI may be complementary methods in the evaluation of primary intraspinal extradural hydatid disease.

Hydatid cyst can be diagnosed by means of the anamnesis if the patient originates from a region where the disease is endemic, or by serological tests. But it is known that the Casoni-Weinberg test is not very reliable [14]. In this present case, the serological tests were negative. Due to the stated clinical history (from Xinjiang province, epidemic area of China) and typical neuroradiological features, spinal hydatid cysts should be considered. The final diagnosis is also confirmed by histopathological examination.

Spinal hydatid disease should be carefully considered when planning a surgery especially in endemic countries. Treatment should be surgical removal without cyst rupture and medical therapy (mebendazole or albendazole) following the surgery. Generally, the major factor influencing the choice of surgical approach is the degree of neuro-foraminal and spinal canal involvement. Antihelminthic drugs should be given for longer periods up to 2 years after surgery [15]. In our case, an anterior approach was adequate for exposure and removal of the lesions. A posterior approach was not considered because we wanted to keep the posterior spinal column mechanically strong and reduced the risk of posterior spread of the infestation. Misdiagnosis of spinal hydatid cyst as tuberculous spondylitis could result in serious consequences. Recurrence (30% 100%) remains a major problem in spinal hydatid disease [16,17]. Long-term hydatid disease caused persistent pain, significant persistent neurologic deficits and spinal instability and resulted in a high morbidity and mortality and poor prognosis. Albendazole treatment should be started in the postoperative stage, preventing late recurrences.

## Conclusion

Thoracic intraspinal extradural hydatid cyst has only occasionally been reported in the literature. Although CT and MRI have developed the diagnosis of both tuberculous spondylitis and hydatid cysts, the two entities could still difficult to be differentiated. Familiarity with typical imaging appearances of complicated hydatid disease in an unusual location may be valuable in making a correct diagnosis and treatment.

## List of abbreviations

CSF: Cerebrospinal fluid
CT: Computerised Tomography
MRI: Magnetic Resonance Imaging
